# Improvements in visual acuity and macular morphology following cessation of anti-estrogen drugs in a patient with anti-estrogen maculopathy resembling macular telangiectasia type 2: a pathogenic hypothesis

**DOI:** 10.1186/s12886-019-1280-1

**Published:** 2019-12-30

**Authors:** Akihiro Shinkai, Wataru Saito, Yuki Hashimoto, Susumu Ishida

**Affiliations:** 1 Department of Ophthalmology, Faculty of Medicine and Graduate School of Medicine, Hokkaido University, Nishi 7, Kita 15, Kita-ku, Sapporo, 060-8638 Japan; 2 Kaimeido Eye and Dental Clinic, Sapporo, Japan

**Keywords:** Macular telangiectasia type 2, Photoreceptor, Tamoxifen, Retinal pigment epithelium, Toremifene, Toxicity

## Abstract

**Background:**

The relationship between anti-estrogen drugs and macular telangiectasia type 2 (MacTel-2) remains unknown. Here we report a case with anti-estrogen maculopathy resembling MacTel-2 with improved visual function and macular morphology following cessation of anti-estrogen drugs.

**Case presentation:**

A 53-year-old woman presented with a 5-month history of central vision loss and anorthopia in both eyes. She had received oral tamoxifen followed by toremifene for 69 months. Funduscopy, fluorescein angiography, and optical coherence tomography (OCT) revealed MacTel-2-like findings OU. Fundus autofluorescence (FAF) showed hyper-autofluorescence at the fovea OU. Visual acuity, macular morphology on OCT, and FAF findings gradually improved after cessation of anti-estrogen drugs.

**Conclusions:**

In the present case, visual acuity, macular morphology, and impairment of the retinal pigment epithelium (RPE) improved following cessation of anti-estrogen drugs, suggesting the relationship between retinal toxicity of anti-estrogen drugs and the development of MacTel-2-like findings. From these results and the previous observations, toxicity of both photoreceptor and RPE cells caused by anti-estrogen drugs may contribute to the development of anti-estrogen maculopathy similar to MacTel-2.

## Background

Anti-estrogen drugs are widely used to treat hormone-dependent breast cancer. Tamoxifen and toremifene rarely cause toxic retinopathy, characterized by intraretinal crystals and changes in retinal pigment epithelium (RPE) [1, 2]. Anti-estrogen drug-associated retinopathy has been reported to result basically from use of tamoxifen, as called tamoxifen retinopathy; however, there were few reports on toremifene use [3]. Treatment strategy for tamoxifen retinopathy is cessation of the drug. Visual function may stabilize after treatment but commonly be unrecoverable [4].

Findings such as intraretinal crystals, RPE abnormality, and foveal hypo-reflective space on optical coherence tomography (OCT), as seen in patients with tamoxifen retinopathy, may also be observed in patients with macular telangiectasia type 2 (MacTel-2), showing similarity of clinical findings between the two diseases [5]. A previous study of 17 cases with medical history of tamoxifen administration reported MacTel-2-like findings [5–7]. However, the relationship between anti-estrogen drugs and MacTel-2 is poorly understood.

We herein report a case with anti-estrogen maculopahty resembling MacTel-2 showing improved visual acuity and macular morphology following cessation of anti-estrogen drugs.

## Case presentation

A 53-year-old woman presented with a 5-month history of central vision loss with anorthopia of both eyes. The patient received oral tamoxifen 20 mg/day for 58 months (cumulative dose; 34.8 g) and thereafter toremifene 40 mg/day for 11 months (cumulative dose; 13.2 g) for breast cancer; however, toremifene was discontinued several days prior to her first hospital visit. She had no significant personal or family medical history, except for breast cancer.

**Fig. 1:**
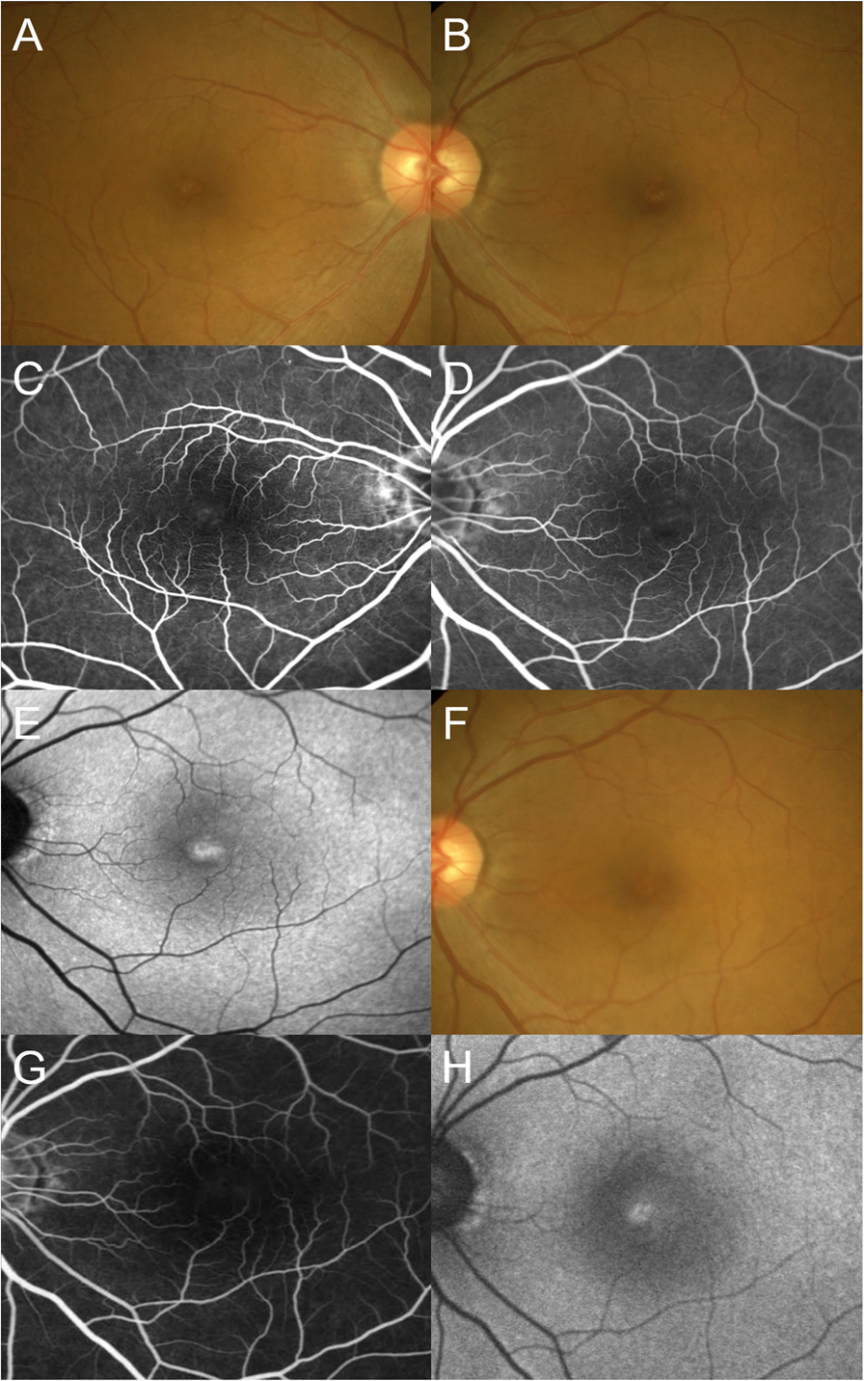
Photographs of right eye (**a**, **c**) and left eye (**b**, **d**, **e**-**h**) in a 53-year-old woman with macular telangiectasia type 2-like findings. **a**, **b** Fundus images showing foveal depigmentation of the retinal pigment epithelium (RPE) and the surrounding ringed gray-colored reduced retinal transparency in both eyes at the initial visit. **c**, **d** Late-phase fluorescein angiography showing diffuse dilated retinal capillaries at the macula with mild leakage at the fovea at the initial visit. **e** Fundus autofluorescence (FAF) showing hyper-autofluorescence at the fovea at the initial visit. **f** Fourteen months after the initial visit, RPE depigmentation at the fovea decreased. **g** Twenty-five months after the initial visit, the extent of foveal vascular leakage on late-phase fluorescein angiography decreased. **h** Thirty-one months after the initial visit, the size of hyper-autofluorescence on FAF shrunk

**Fig. 2:**
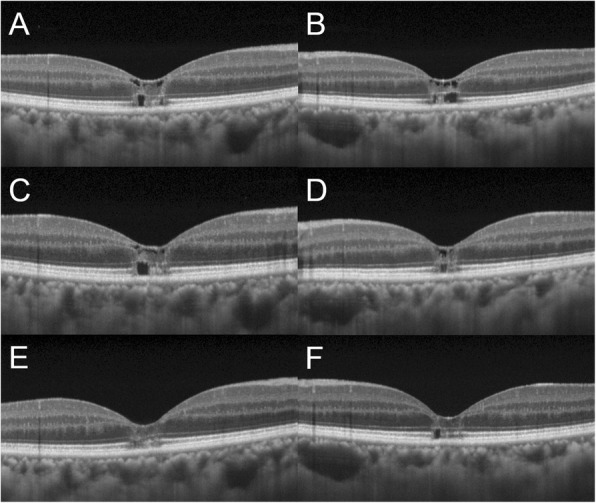
Sequential changes in horizontal images through the fovea of enhanced depth imaging optical coherence tomography in the right (**a**, **c**, **e**) and left (**b**, **d**, **f**) eyes. **a**, **b** At the initial visit, the loss of ellipsoid zone (EZ), interdigitation zone, and inner and outer lamellar cavities at the fovea were observed in both eyes. **c**, **d** Three months after the initial visit, the area of the EZ loss and the extents of the inner and outer lamellar cavities were reduced in the left eye but not in the right eye. **e**, **f** Twenty-two months after the initial visit, the area of EZ loss further decreased in both eyes compared with that at 3 months after the initial visit, with the resolution of the inner lamellar cavity and almost resolution of the outer lamellar cavity in both eyes. However, the loss of the interdigitation zone at the fovea persisted

Her best-corrected visual acuity (BCVA) was 0.4 OD and 0.3 OS with mild myopia. The cornea and lens were clear OU. Funduscopic examination revealed a de-pigmented lesion of the RPE at the fovea, surrounded by a gray-colored lesion OU (Fig. 1a, b). Intraretinal crystals and pigmentation were not observed OU. Initial fluorescein angiography revealed dilatation of macular retinal capillaries with mild late dye leakage OU (Fig. 1c, d). Enhanced depth imaging OCT (EDI-OCT) revealed loss of the ellipsoid zone (EZ) and interdigitation zone (IZ) OU, inner and outer lamellar cavities OU at the macula, all of which were part of typical MacTel-2 findings (Fig. 2a, b). Fundus autofluorescence (FAF) demonstrated hyper-autofluorescence at the fovea OU (Fig. 1e). The patient was followed up with no treatment.

Three months after the first visit, her BCVA spontaneously improved to 0.9 OD and 0.6 OS and the area of the EZ loss and the extent of the inner and outer lamellar cavities on EDI-OCT were reduced OS and remained unchanged OD (Fig. 2c, d). She received sub-Tennon (40 mg) and intravitreal (4 mg) injections of triamcinolone acetonide OU at 3 and 6 months after her first visit, respectively. Fourteen months after the first visit, BCVA improved to 1.0 OD and 0.7 OS, with improvement of the macular morphology OU. On funduscopy, the extents of the foveal RPE depigmentation also decreased OU (Fig. 1f). Twenty-two months after the first visit, the area of EZ loss further decreased OU, with the resolution of the inner lamellar cavity and near-resolution of the outer lamellar cavity OU (Fig. 2e, f). However, the loss of the foveal IZ persisted. Twenty-five months after the first visit, dilated perifoveal retinal capillaries on fluorescein angiography showed almost no dilatation, with no late leakage OU (Fig. 1g). Thirty-one months after the first visit, the BCVA was 1.2 OD and 0.7 OS. The macular morphology remained unchanged OU. The hyper-autofluorescent area on FAF subsided but persisted (Fig. 1h).

## Discussion and conclusions

We encountered an anti-estrogen maculopathy case exhibiting part of typical MacTel-2 findings. Use of toremifene and marked improvements of the BCVA and macular morphology following cessation of anti-estrogen drugs were rarely described in the literature.

Clinical findings in the present case resembled those of MacTel-2 at the early stage except for the absence of dye leakage at the temporal side of the macula on fluorescein angiography. In patients with MacTel-2, Müller cell dysfunction has been proposed based on histopathological examination [8], as being observed as inner and outer lamellar cavities at the fovea on OCT. However, the mechanism causing the Müller cell abnormality remains unresolved. Patients with MacTel-2 at the initial stage have disruptions of the EZ despite the lack of foveal inner and outer lamellar cavities [9]. Moreover, multimodal imaging in patients with MacTel-2 of very early stage revealed a decrease of cone density even when macular EZ was intact [10]. These observations suggest photoreceptor damage prior to Müller cell impairment. Interestingly, a study published very recently have demonstrated elevated levels of cytotoxic deoxysphingolipids following a decrease in serum serine levels in MacTel-2 patients with idiopathic etiology as well as gene mutation of serine metabolism [11]. Deoxysphingolipids induced photoreceptor apoptosis in human retinal organoids and mice supplemented with serine diet showed decreased photopic response on electroretinography [11]. These results indicate that abnormality of sphingolipid metabolism plays a role in the pathogenesis of the photoreceptor loss observed in patients with MacTel-2. Importantly, it has been reported that tamoxifen suppresses sphingolipid metabolism [12]. Therefore, chronic retinal damage following tamoxifen-induced impairment of sphingolipid metabolism may be involved in the pathogenesis of MacTel-2-like findings associated with patients taking tamoxifen.

In mice with serine diet, elevated deoxysphingolipids were observed in the RPE as well. The elevation of the cytotoxic metabolites is theorized to affect the RPE [11]. Actually, RPE impairment on FAF occurs prior to OCT and angiographic changes in MacTel-2 [13], suggesting impairment of the RPE preceding abnormalities of Müller cells and photoreceptors. In the present case, hyper-autofluorescence on FAF and the loss of foveal IZ on OCT persisted despite restored macular EZ following cessation of anti-estrogen drugs, suggesting persistent RPE dysfunction. Moreover, tamoxifen and toremifene cause RPE cell death by inhibiting phagocytosis of the rod’s outer segments by RPE cells due to lysosomal destabilization [14, 15]. Bietti’s crystalline dystrophy, associated with intraretinal crystals as seen in MacTel-2, is caused by lysosomal dysfunction following accumulation of free cholesterol in the RPE cells as a result of gene mutation [16]. Thus, tamoxifen-induced adverse effects on sphingolipid metabolism and lysosomal function in RPE cells may be involved in the development of MacTel-2-like findings in patients taking tamoxifen.

Little is known about tamoxifen’s effect on Müller cells. Tamoxifen-induced abnormality of sphingolipid metabolism may possibly affect Müller cells as well as photoreceptor cells. Next, a recent study with a model of photoreceptor degeneration revealed that loss of rod cells was simultaneously associated with damage to the neurovascular unit comprising photoreceptor, RPE, and Müller glial cells [17], suggesting a coexisting interaction between the photoreceptor/RPE and Müller glia. Therefore, impairment of Müller cells may parallel photoreceptors/RPE cytotoxicity caused by anti-estrogen drugs.

The effectiveness of intravitreal injections of triamcinolone acetonide (IVTA) has been reported in a case with tamoxifen retinopathy and an animal model of MacTel-2 [18, 19]. Therefore, we performed IVTA for the present case. Although the BCVA and macular morphology further improved after administration of IVTA, it is difficult to assess whether the treatment led to improvement of the macular morphology.

Toremifene is structurally and pharmacologically similar to tamoxifen. A previous study showed that there was no significant difference in frequency of retinopathy between patients receiving tamoxifen and toremifene three years after the start of the drugs [3]. Therefore, it is difficult to determine whether which drug caused retinal toxicity in the present case. Tamoxifen retinopathy usually appears to occur when patients received total cumulative dose of greater than 100 g [1]. The total cumulative dose administered in our case was much less than 100 g. This may be one reason why photoreceptors recovered in this case.

In conclusion, BCVA, macular morphology, and impairment of the RPE improved following cessation of anti-estrogen drugs in an anti-estrogen maculopathy case with MacTel-2-like findings. These results suggest the relationship between retinal toxicity of anti-estrogen drugs and the development of findings resembling MacTel-2 in the present case.

## Data Availability

All data generated or analyzed during this study are included in this published article.

## Abbreviations

BCVA: Best-corrected visual acuity
EDI-OCT: Enhanced depth imaging optical coherence tomography
EZ: Ellipsoid zone
FAF: Fundus autofluorescence
IVTA: Intravitreal injections of triamcinolone acetonide
IZ: Interdigitation zone
MacTel-2: Macular telangiectasia type 2
RPE: Retinal pigment epithelium

## References

[1] Nayfield SG, Gorin MB (1996). Tamoxifen-associated eye disease. Rev J Clin Oncol.

[2] Tang R, Shields J, Schiffman J, Li H, Locher D, Hampton J (1997). Retinal changes associated with tamoxifen treatment for breast cancer. Eye (Lond).

[3] Parkkari M, Paakkala AM, Salminen L, Holli K, Finnish Breast Cancer Group (2003). Ocular side-effects in breast cancer patients treated with tamoxifen and toremifene: a randomized follow-up study. Acta Ophthalmol Scand.

[4] Rahimy E, Sarraf D (2012). Bevacizumab therapy for tamoxifen-induced crystalline retinopathy and severe cystoid macular edema. Arch Ophthalmol.

[5] Wolf-Schnurrbusch UEK, Leung I, Sallo FB, Clemons TE, Chew EY, Bird AC, MacTel Study Group (2018). Potential effects of hormone therapy in type 2 idiopathic macular telangiectasia. Ophthalmic Res.

[6] Doshi RR, Fortun JA, Kim BT, Dubovy SR, Rosenfeld PJ (2014). Pseudocystic foveal cavitation in tamoxifen retinopathy. Am J Ophthalmol.

[7] Behrens A, Sallam A, Pemberton J, Uwaydat S (2018). Tamoxifen use in a patient with idiopathic macular telangiectasia type 2. Case Rep Ophthalmol.

[8] Powner MB, Gillies MC, Tretiach M, Scott A, Guymer RH, Hageman GS (2010). Perifoveal muller cell depletion in a case of macular telangiectasia type 2. Ophthalmology.

[9] Maruko I, Iida T, Sekiryu T, Fujiwara T (2008). Early morphological changes and functional abnormalities in group 2A idiopathic juxtafoveolar retinal telangiectasis using spectral domain optical coherence tomography and microperimetry. Br J Ophthalmol.

[10] Charbel Issa P, Heeren TF, Kupitz EH, Holz FG, Berendschot TT (2016). Very early disease manifestations of macular telangiectasia type 2. Retina.

[11] Gantner ML, Eade K, Wallace M, Handzlik MK, Fallon R, Trombley J (2019). Serine and lipid metabolism in macular disease and peripheral neuropathy. N Engl J Med.

[12] Morad SA, Cabot MC (1851). Tamoxifen regulation of sphingolipid metabolism--therapeutic implications. Biochim Biophys Acta.

[13] Wong WT, Forooghian F, Majumdar Z, Bonner RF, Conningham D, Chew EY (2009). Fundus autofluorescence in type 2 idiopathic macular telangiectasia: correlation with optical coherence tomography and microperimetry. Am J Ophthalmol.

[14] Mannerström M, Mäenpää H, Toimela T, Salminen L, Tähti H (2001). The phagocytosis of rod outer segments is inhibited by selected drugs in retinal pigment epithelial cell cultures. Pharmacol Toxicol.

[15] Kim LA, Amarnani D, Gnanaguru G, Tseng WA, Vavvas DG, PA D’A (2014). Tamoxifen toxicity in cultured retinal pigment epithelial cells is mediated by concurrent regulated cell death mechanisms. Invest Ophthalmol Vis Sci.

[16] Hata M, Ikeda HO, Iwai S, Iida Y, Gotoh N, Asaka I (2018). Reduction of lipid accumulation rescues Bietti's crystalline dystrophy phenotypes. Proc Natl Acad Sci U S A.

[17] Ivanova E, Alam NM, Prusky GT, Sagdullaev BT (2019). Blood-retina barrier failure and vision loss in neuron-specific degeneration. JCI Insight.

[18] Jeng KW, Wheatley HM (2015). Intravitreal triamcinolone acetonide treatment of tamoxifen maculopathy with associated cystoid macular edema. Ret Cases Brief Rep.

[19] Shen W, Lee SR, Araujo J, Chung SH, Zhu L, Gillies MC (2014). Effect of glucocorticoids on neuronal and vascular pathology in a transgenic model of selective Müller cell ablation. Glia.

